# Super Enhancer Driven Hyaluronan Synthase 3 Promotes Malignant Progression of Nasopharyngeal Carcinoma

**DOI:** 10.7150/jca.83954

**Published:** 2023-06-12

**Authors:** Quanzhu Chen, Qian Peng, Jing Cai, Ying Liu, Xingxing Lu, Wei Xiong, Zhaoyang Zeng, Guiyuan Li, Xiaoling Li, Xiayu Li, Bo Xiang, Mei Yi, Pan Chen

**Affiliations:** 1 Hunan Cancer Hospital and the Affiliated Cancer Hospital of Xiangya School of Medicine, Central South University, Changsha 410013 Hunan, China.; 2 The Key Laboratory of Carcinogenesis of the Chinese Ministry of Health, Cancer Research Institute and School of Basic Medical Sciences, Central South University, Changsha 410008 Hunan, China.; 3 The Key Laboratory of Carcinogenesis and Cancer Invasion of the Chinese Ministry of Education, Cancer Research Institute and School of Basic Medical Sciences, Central South University, Changsha 410078 Hunan, China.; 4 Hunan Key Laboratory of Nonresolving Inflammation and Cancer, The Third Xiangya Hospital, Central South University, Changsha 410013 Hunan, China.; 5 Department of Pathology, the Second Xiangya Hospital, Central South University, Changsha 410000 Hunan, China.; 6 Department of Dermatology; National Clinical Research Center for Geriatric Disorders, Xiangya Hospital, Central South University, Changsha 410008 Hunan, China.

**Keywords:** nasopharyngeal carcinoma, super-enhancer, hyaluronan, hyaluronan synthase 3, EGFR/AKT/ERK signaling pathway

## Abstract

Nasopharyngeal carcinoma (NPC) is a malignant tumor of the head and neck with high metastatic and invasive nature. Super enhancers (SEs) control the expression of cell identity genes and oncogenes during tumorigenesis. As a glycosaminoglycan in the tumor microenvironment, hyaluronan (HA) is associated with cancer development. High expression of hyaluronan synthase 3 (HAS3) resulted in HA deposition, which promoted the growth of cancer cell. However, its role in NPC development remains elusive. We demonstrated that the levels of HAS3 mRNA or protein were increased in NPC cell lines. Transcription of HAS3 is associated with SE. Disruption of SE by bromodomain containing 4 (BRD4) inhibitor JQ1 resulted in downregulation of HAS3 and inhibition of cell proliferation and invasiveness in NPC cells. Inhibition of HA synthesis by HAS inhibitor 4-MU suppressed cell growth and invasion of NPC cells, whereas HA treatment exerted opposite effects. Genetically silencing HAS3 in HK1 and FaDu NPC cells attenuated cell proliferation and mobility, while re-expression of HAS3 enhanced malignant potential of CNE1 and CNE2 NPC cells. Furthermore, loss of HAS3 impaired metastatic potential of HK1 cells in nude mice. Mechanistically, inhibition of HA synthesis by chemical inhibitor or silencing HAS3 led to reduction of the levels of phosphorylation of EGFR, AKT, and ERK proteins. In contrast, exogenous HA treatment or forced expression of HAS3 activated EGFR/AKT/ERK signaling cascade. This study suggested that HAS3 is driven by SE and overexpressed in NPC. High expression of HAS3 promotes the malignant features of NPC via activation of EGFR/AKT/ERK signaling pathway.

## Introduction

Nasopharyngeal carcinoma (NPC) is a malignant tumor of head and neck cancer occurring in the top and lateral walls of the nasopharyngeal cavity. Owing to this unique anatomical structure of nasopharyngeal cavity, NPC can be difficult to make an early diagnosis 1. In addition, NPC is characterized by high invasion and early metastasis, 70-80% of NPC patients have developed cervical lymph node metastases at the first diagnosis 2-5. However, the underlying mechanism of NPC metastasis is still not clear.

NPC is characterized as having comparatively low mutation rate, widespread hypermethylation, high copy number alterations and chromosome abnormalities by Genome-wide studies 6-14. Compared with inactivation of tumor suppressor genes, tumorigenic mutation is a very rare cause for inherited mutations of NPC, such as the mutation of K-RAS gene or epidermal growth factor receptor (EGFR) gene 8, 15, 16. Furthermore, it has been demonstrated that chromatin remodeling factors, such as ARID1A, MLL2, and MLL3 have shown high mutation frequency in NPC 8, 16. Thus, the epigenetic alterations may play a crucial role in NPC initiation and development. It will be highly desirable if we study the activation of oncogenes in the processes evolve on NPC from the perspective of epigenetics.

Super enhancers (SEs) are related to epigenetic regulatory mechanisms, which involve in the regulation of gene expression 17. As the large clusters of cis-elements, SEs are characterized by intensive H3K27ac modification, and significantly enriched with the master transcription factors (TFs) and mediator. Compared with the typical enhancers (TEs), SEs possess stronger capability of driving genes expression, and are highly sensitive to JQ1 (a SE inhibitor) 18. In many types of cancer, SEs act as key oncogenic drivers during tumorigenesis 19-21. Therefore, in NPC development, understanding the role of the SEs landscape will provide a novel approach for identifying oncogenic factors. Through SE profile and RNA-Seq, we found hyaluronan synthase 3 (HAS3) was driven by SE.

As a core mucopolysaccharide component of extracellular matrix (ECM), hyaluronan (HA) involves in many biological processes, such as malignant cell migration 22-24, and also promotes the proliferation and tumorigenicity of CNE2 cell lines 25. HA is synthesized by three hyaluronan synthase (HAS) enzymes, HAS1, HAS2, and HAS3 26. High expression of HAS3 results in HA deposition, which induces extracellular matrix remodeling and promotes the growth of colon, prostate and pancreatic cancer cell 27-29. However, HAS3 knockdown or inhibition of HA suppresses cell proliferation and survival, decreases esophageal xenograft tumorigenesis, and suppresses epidermal growth factor receptor (EGFR)/AKT/ERK signaling pathway in esophageal squamous cell carcinoma (ESCC) and lung cancer 30, 31.

Previous research has confirmed that the overexpression of EGFR is common in NPC and the EGFR signaling pathway plays the significant role in the pathogenesis of NPC 32, 33. EGFR and downstream signaling proteins affect many cellular functions, such as proliferation, differentiation, migration, and apoptosis 34, 35. It was found that through activating EGFR-SRC signaling, HA and HAS3 could induce oncogenic actions 36-38. However, the precise mechanisms that mediate HAS3-dependent malignant cell migration remain unclear. In this study, we demonstrated that the levels of HAS3 mRNA or protein were increased in NPC cell lines. Inhibition of HA synthesis suppressed cell growth and invasion of NPC cells, whereas HA treatment exerted opposite effects. Genetically silencing HAS3 in NPC cells attenuated cell proliferation, mobility, and invasiveness in vitro, and metastasis in vivo, while re-expression of HAS3 enhanced malignant potential of NPC cells. Mechanistically, inhibition of HA synthesis or silencing HAS3 led to reduction of the levels of phosphorylation of EGFR, AKT and ERK proteins. In contrast, exogenous HA treatment or forced expression of HAS3 activated EGFR/AKT/ERK signaling cascade. This study suggested that HAS3 is driven by SE and overexpressed in NPC. High expression of HAS3 promotes the malignant features of NPC via activation of EGFR/AKT/ERK signaling pathway.

## Material and Methods

### Cell lines and cell culture

NP69, HK1, C666-1, CNE1, CNE2, and FaDu cell lines were used in this study. NP69 is an immortalized nasopharyngeal epithelial cell line, which maintained in our lab 39. The human NPC cell lines HK1, C666-1, CNE1 and CNE2 are maintained in our lab 40-43. FaDu is a hypopharyngeal carcinoma cell line purchased from the National Infrastructure of Cell Line Resource (Shanghai, China) 44, 45. All these cells were cultured in 10% fetal bovine serum (FBS, Gibco, Grand Island, NY, USA), RPMI 1640 medium (Gibco, Grand Island, NY, USA) at 37℃, 5% CO_2_ 46.

### RNA isolation and real-time reverse-transcription PCR (qPCR)

Total cellular RNAs were extracted using Trizol reagent (Life Technologies, Grand Island, NY, USA). cDNAs were synthesized from the RNAs using RNase-free DNase I (Takara, Beijing, China) and RevertAid First Strand cDNA Synthesis Kits (Thermo Fisher Scientific, Beijing, China). Quantitative PCR mixtures were prepared according to the manufacturer's manual of SYBR Green (Bimake, Shanghai, China) and reactions were run in a CFX96 Touch™ Real-Time PCR Detection System (Bio-Rad, Richmond, CA, USA) 47. 2-ΔΔCT methods were adopted for genes relative expression levels. Primers sequences are as follows: HAS3 forward primer ACTACATCCAGGTGTGCGAC; HAS3 reverse primer CAGCCAAAGTAGGACTGGCA. GAPDH forward primer AACGGATTTGGTCGTATTGG; GAPDH reverse primer TTGATTTTGGAGGGATCTCG.

### Western blotting

Western blotting was performed to measure protein expression levels. NPC cells were lysed with RIPA buffer (Beyotime, Jiangsu, China). Equal cellular protein sample amounts from different extracts were separated by 10% SDS-PAGE gels and transferred onto PVDF membranes (Millipore, Billerica, MA, USA) 48. After transfer, membranes were blocked with 5% non-fat milk in Tris buffer saline containing 0.1% Tween-20 for 1 hour at room temperature and then incubated with primary antibodies at 4 °C overnight. The following antibodies against HAS3, p-EGFR (Y992), p-AKT (S473), p-ERK1/2 (ABclonal Technology, Woburn, MA, USA), EGFR (Proteintech, Chicago, IL, USA), AKT, ERK1/2 (Cell Signaling Technology, Beverly, MA, USA) and GAPDH (ABclonal Technology) were used.

### Cell viability assay

Cell Counting Kit-8 (CCK-8, Beyotime) was performed as previously described 49, 50. Briefly, NPC cells (1 × 10^3^ cells/mL) were plated into a 96-well plate in each well in quintuplicate and treated with different dose of JQ1, 4-Methylumbelliferone (4-MU, Selleck Chemicals, Houston, TX, USA), or HA (8000 Da, Sigma-Aldrich, St Louis, MO, USA) for 24 h. Cells were incubated with 10 μl of CCK8 for 2h at 37℃. The absorbance of sample was measured at 450 nm using a microplate reader. Data were presented as means ± SD from five wells per experiment.

### Cell migration and invasion assays

Tumor cell migration and invasion were determined by Transwell (Corning‐Costar, Cambridge, MA, USA) migration and invasion assays as previously described 42. In serum-free medium, cell suspensions were seeded onto 8-μm-pore Transwell upper inserts precoated without (migration assay) or with (invasion assay) 15 μL Matrigel (BD Biosciences, Bedford, MA, USA) at a density of 10^5^ cells/well, and then the inserts were held in a lower chamber with 700 μl of RPMI 1640 medium containing 15% FBS. After culturing for 10-48 h at 37℃, cells that migrated to the lower surface of the membrane were fixed and stained with 0.1% crystal violet as previously described 45, 51. The number of tumor cells were counted in five random fields to calculate the average number of cells.

### Stable knockdown and overexpression experiments

For knockdown experiments, tumor cells were infected with shRNA (GenePharma Co., Ltd., Shanghai, China) expressing lentivirus and selected by puromycin. For stable overexpression, tumor cells were infected with HAS3 expressing lentivirus and selected by puromycin. Sequences of shRNAs are as follows: shHAS3#1 GCTCTACAACTCTCTGTGGTTC; shHAS3#2 CCATTGCTACCATCAACAAAT.

### In vivo metastasis study

The animal protocols used were reviewed and approved by the ethical review committee of The Central South University of China. The animal experiment followed ARRIVE guidelines. The suspended cells (1 × 10^6^/0.2 mL) were injected into the lateral tail vein of 5-week-old male BALB/c nude mice (Hunan Slack King Laboratory Animal Co. Ltd., Changsha, China), and they were sacrificed 6 weeks after tumor cell inoculation 52. Lung tissues were fixed in 4% saline-buffered formalin, embedded in paraffin, sectioned at 5 μm, and then stained with hematoxylin & eosin (H&E) as previously described 53. A minimum of 15 sections was examined per mouse under a light microscope.

### Statistical analysis

SPSS v17.0 software (SPSS, Chicago, IL, USA) was employed for statistical analysis, and Prism 5.0 (Graphpad Software, CA, USA) was used for Statistical histograms. Student's t-test, Pearson's χ^2^ test, two-way ANOVA were used. Experiments were repeated in triplicate. Significance was defined as *P* < 0.05 (*), *P* < 0.01 (**), or *P* < 0.001 (***).

## Results

### HAS3 is highly expressed in HNSC and correlates with poor survival

**Figure 1:**
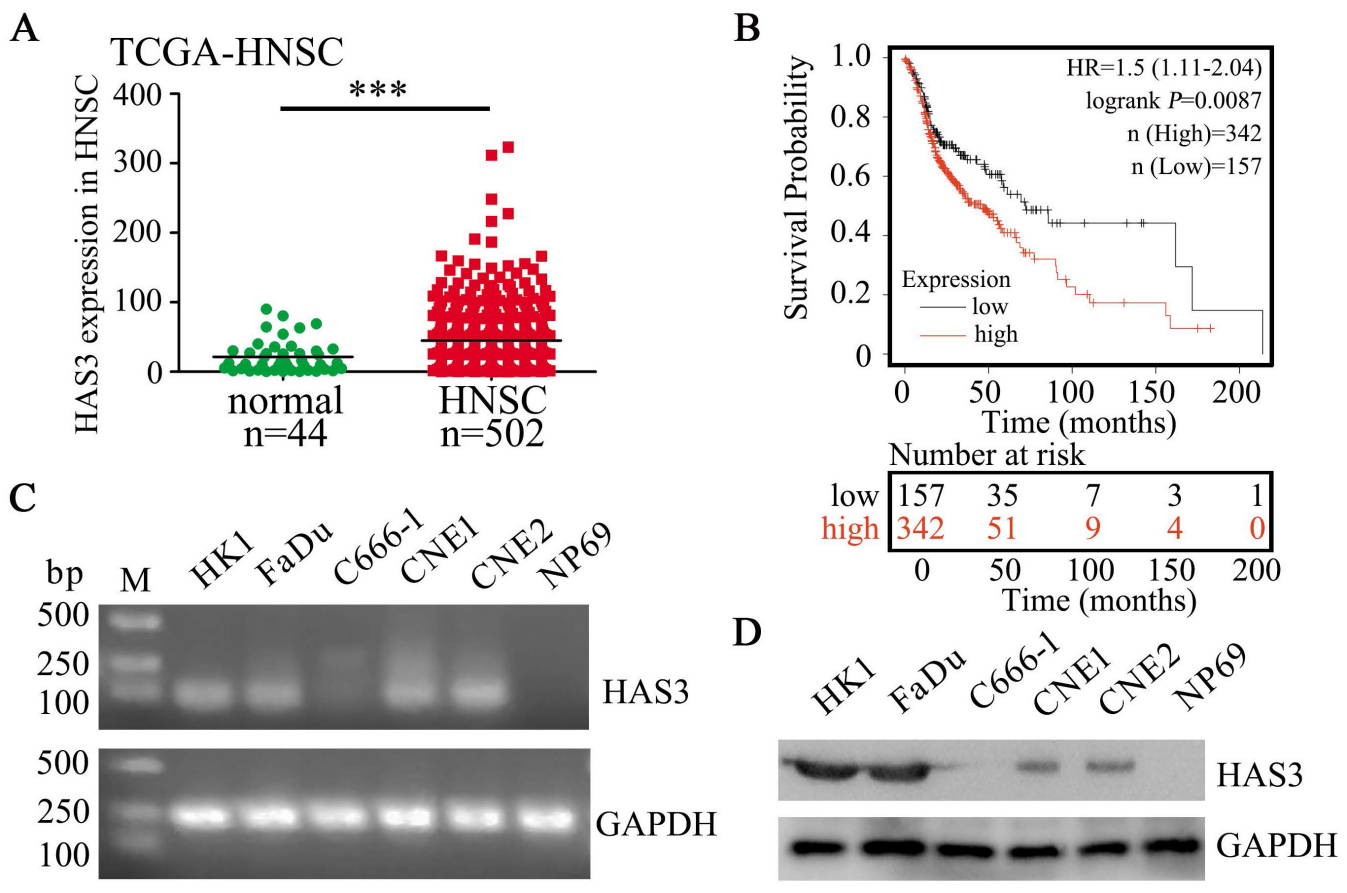
** HAS3 was highly expressed and predicted poor survival in HNSC.** (A) Relative expression of HAS3 in HNSC tissues in comparison with adjacent non‐tumour tissues. (B) Kaplan-Meier overall survival curves of HNSC patients based on HAS3 expression (low vs. high, log‐rank test). Kaplan-Meier plots were generated using Kaplan-Meier Plotter (http://kmplot.com/). (C) HAS3 mRNA level in various cell lines was determined by RT-PCR. (D) HAS3 protein level in various cell lines was determined by western blotting.

Based on analyzing The Cancer Genome Atlas (TCGA) cohort, HAS3 showed up-regulated in head and neck squamous cell carcinomas (HNSC) samples when compared with normal control (*P* < 0.001, Figure 1A), suggesting it might serve an oncogenic role in HNSC development and progression. In addition, Kaplan-Meier plotter analysis showed that the overall survival of HNSC patients with high HAS3 expression was correlated with poor survival (*P* = 0.0087, Figure 1B). Verification experiment using RT-PCR and western blotting indicated that HAS3 was highly expressed in HK1 and FaDu cells, but not expressed in C666-1 cell and normal nasopharyngeal epithelial cell line NP69 (Figure 1C and D).

### JQ1 disrupted HAS3-SE-associated transcription and inhibited NPC cells proliferation and mobility

**Figure 2:**
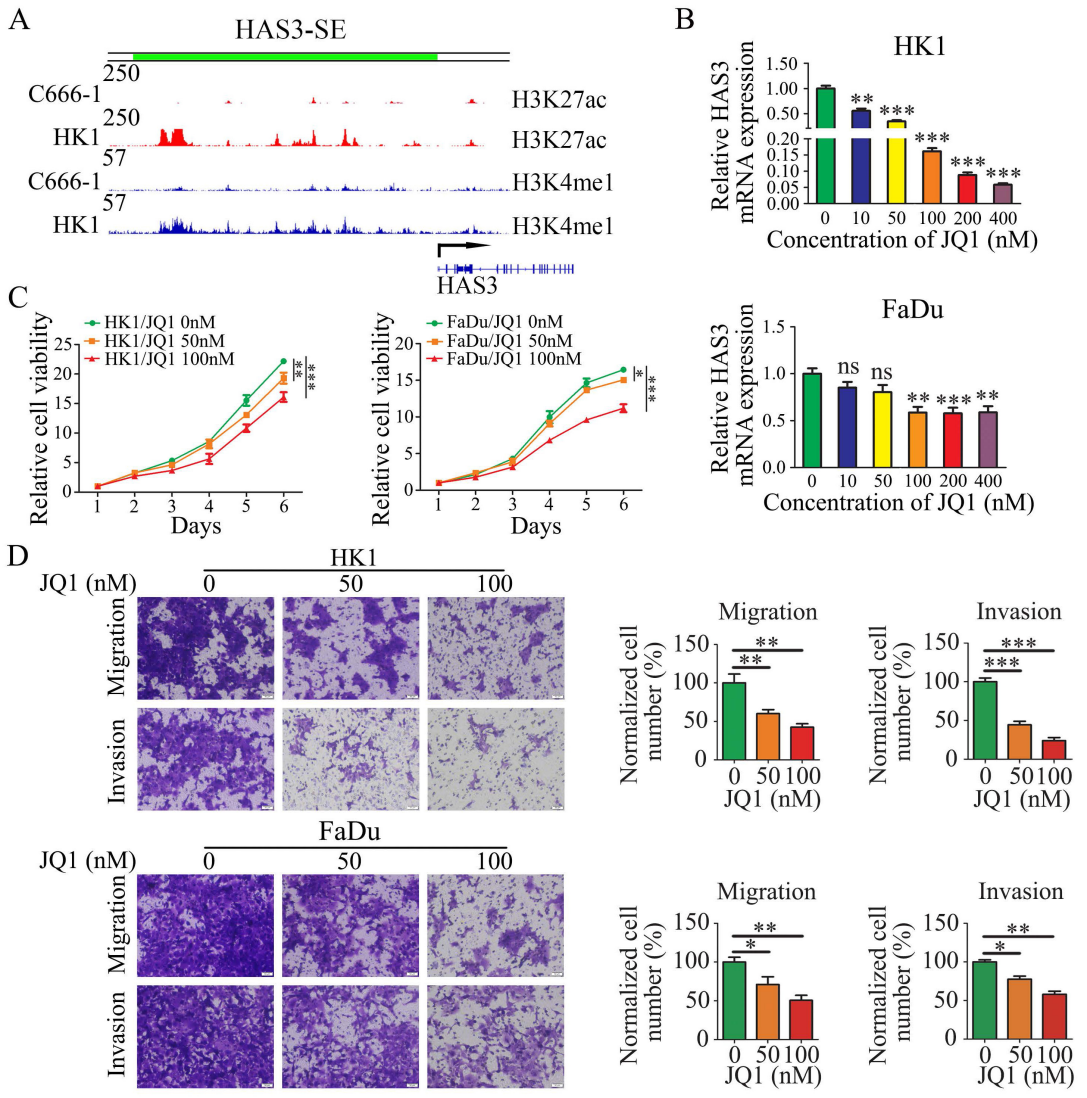
** HAS3 was defined as a SE (HAS3-SE), JQ1 disrupted HAS3-SE-associated transcription and inhibited NPC cells proliferation and invasiveness.** (A) ChIP-seq profiles of H3K4me1, H3K27ac modifications at HAS3-SE in C666-1 and HK1 NPC cells. (B) HAS3 mRNA level in HK1 and FaDu cells was inhibited by JQ1 in a dose-dependent manner. Mean ± SD, n = 3. (C) CCK8 assay showed that JQ1 suppressed the growth of HK1 and FaDu cells. Mean ± SD, n = 3. (D) The migration and invasion of HK1 and FaDu cells treated by JQ1 at various concentrations (× 20). Mean ± SD, n = 3. The control group treated with DMSO. *P < 0.05, **P < 0.01, or ***P < 0.001. ns, no significance.

In previous work (Jing Cai et al. 2019), the SE landscapes and gene transcriptomic between HK1 and C666-1 cells were established by H3K27ac and H3K4me1 ChIP-Seq and RNA-Seq. We defined HAS3 as a SE-driven gene by ROSE algorithm. And we found the region of HAS3 upstream bears high-level H3K4me1 and H3K27ac modifications in HK1 cell lines, but C666-1 cell lines do not present the same modifications (Figure 2A).

As a wide spectrum inhibitor of SE, JQ1 binds to and inhibits the bromodomain containing 4 (BRD4) and preferentially affects coding genes with SEs 54. The BRD4 inhibitor JQ1 has been shown to suppress the tumor cell proliferation 55, 56. Therefore, we examined the effect of JQ1 on the HAS3-SE-associated transcription and NPC cells proliferation. At first, we detected HAS3 mRNA level in HK1 and FaDu cells after treated with JQ1. Quantitative PCR analysis indicated that JQ1 treatment decreased the levels of SE-associated transcript HAS3, in HK1 and FaDu cells in a dose-dependent manner (Figure 2B). Secondly, we compared the proliferation of JQ1 treated cells and control cells. CCK8 assays revealed that JQ1 inhibited the growth of the HK1 and FaDu cell lines in a dose- and time-dependent manner (Figure 2C). Moreover, migration and invasion assays showed that with increasing of dose and time, JQ1 decreased the amount of HK1 and FaDu cells migrated and invaded from the Transwell upper inserts to the lower surface of the membrane significantly (Figure 2D). These results point to the importance of SE-associated transcription for NPC cell proliferation and invasiveness.

### HA promoted NPC cells proliferation, migration, and invasion

**Figure 3:**
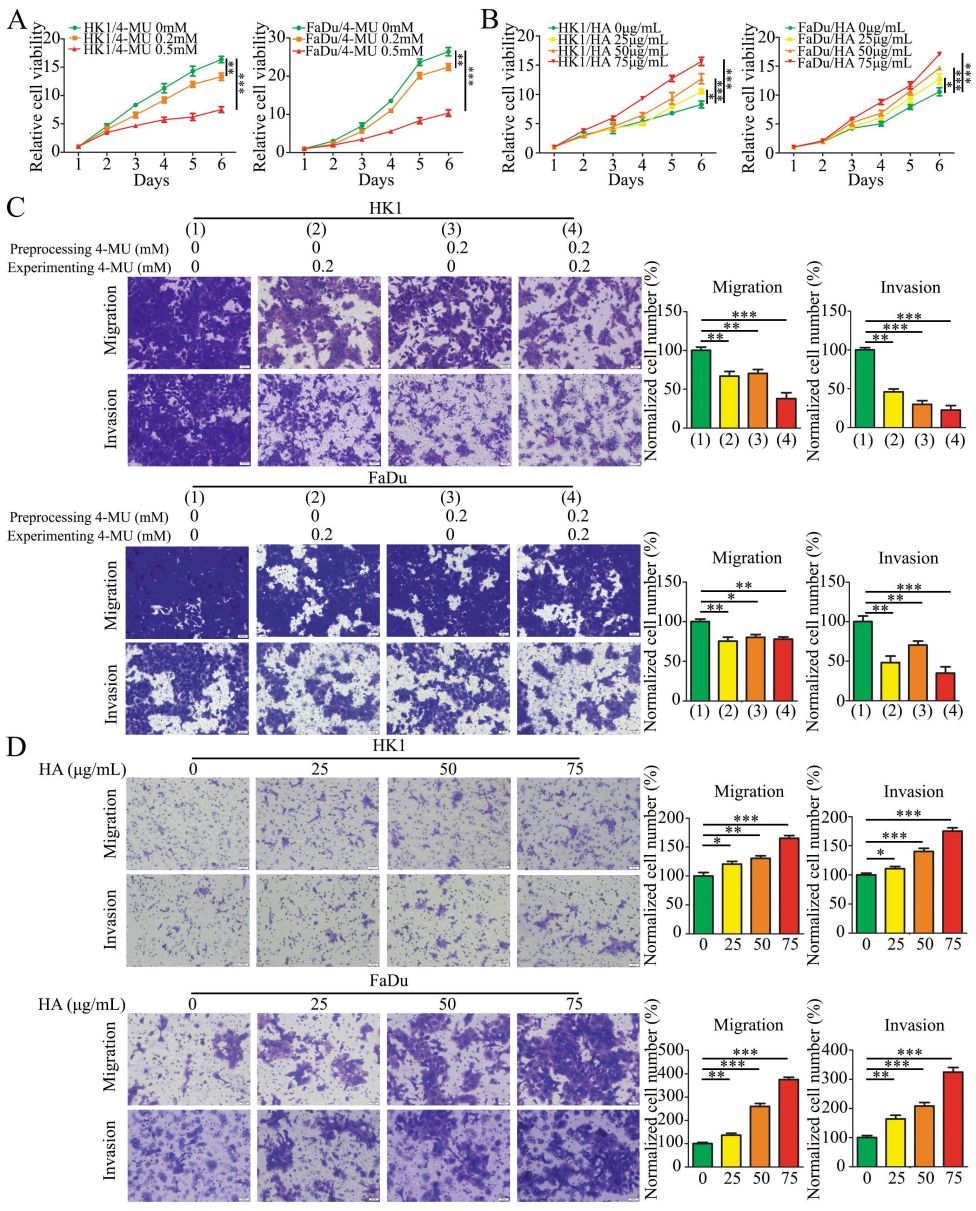
** HA promoted NPC cells proliferation, migration, and invasion.** (A and B) CCK8 assay showed that 4-MU (A) inhibited cell growth, while HA (B) promoted cell growth. Mean ± SD, n = 3. (C) The migration and invasion of HK1 and FaDu cells treated by 4-MU pre-cell seeding or/and post-cell seeding (× 20). Mean ± SD, n = 3. (D) The migration and invasion of HK1 and FaDu cells treated by HA with different concentrations (× 20). Mean ± SD, n = 3. *P < 0.05, **P < 0.01, or ***P < 0.001.

To examine whether the HA promotes the malignant biological behaviors of NPC cells, we tested the effects of HA and 4-MU by CCK-8 and Transwell migration and invasion assays. 4-MU is an HA synthesis inhibitor that acts by depleting a common substrate for HASs, the cellular UDP-glucuronic acid (UDP-GlcUA). The effects of 4-MU on cell proliferation were shown that 4-MU inhibited cell proliferation in a concentration-dependent manner in HK1 and FaDu cells (Figure 3A). In addition, HK1 and FaDu cells were treated with 4-MU at 0.2 mM concentration before or/and during the migration and invasion assays experiment. The results showed that 4-MU suppressed the amount of HK1 and FaDu cells migration and invasion (Figure 3C). On the contrary, the results for HA were completely different. The growth of HK1 and FaDu cells was significantly promoted by HA in a concentration-dependent manner (Figure 3B). We also demonstrated that HA in media promoted additional migration and invasion in a concentration-dependent manner of HK1 and FaDu cells (Figure 3D).

### HAS3 promoted malignant potential of NPC cell in vitro

**Figure 4:**
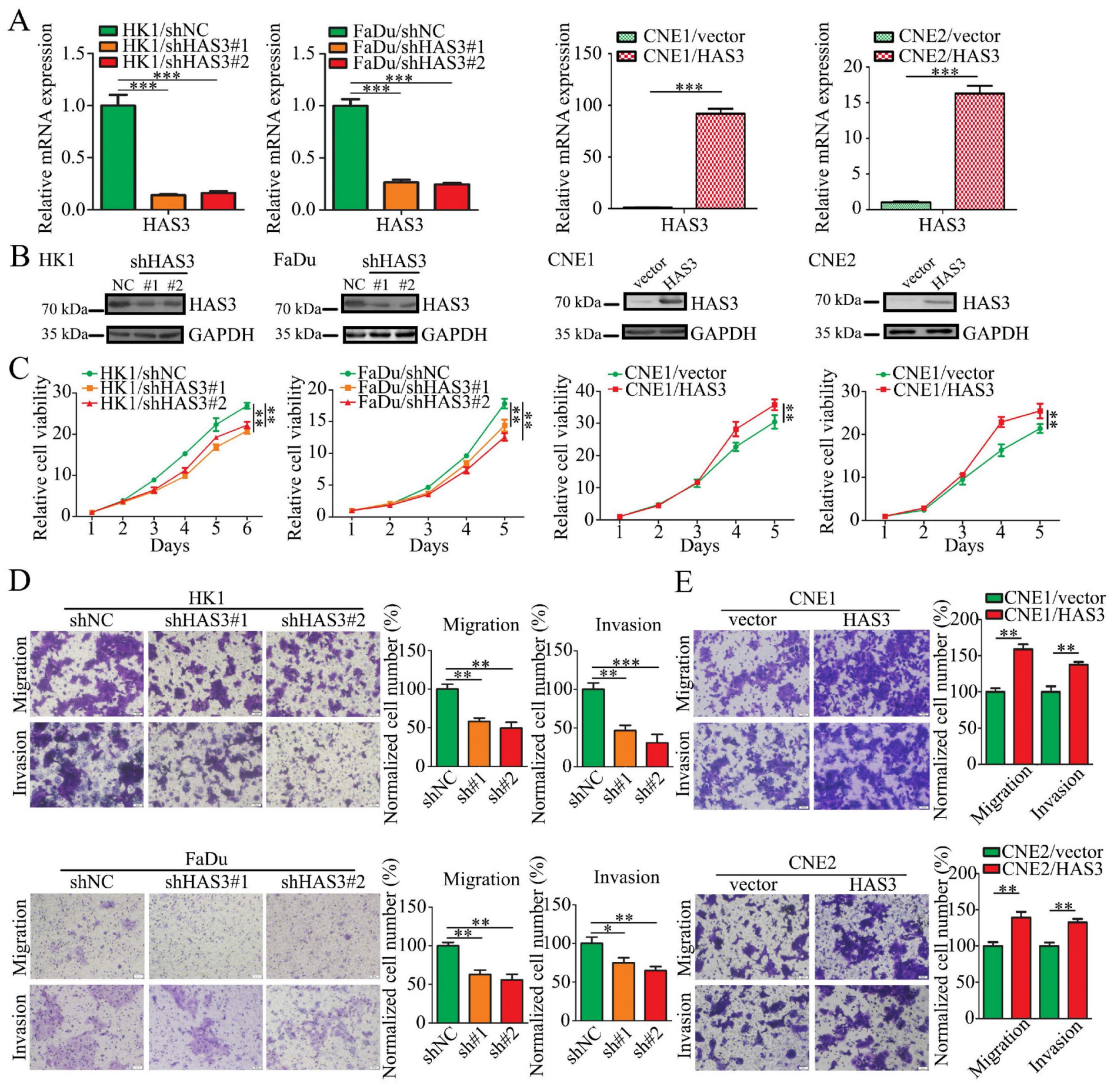
** Loss or gain of function of HAS3 in NPC cell.** (A) The mRNA levels of HAS3 in loss-of-function or gain-of-function NPC cell models were measured using real-time qPCR. Mean ± SD, n = 3. (B) The protein levels of HAS3 in loss-of-function or gain-of-function NPC cell models were measured using western blotting. (C) Cell growth and proliferation measured using CCK8 assay. Mean ± SD, n = 3. (D and E) Cell migration and invasion measured using Transwell migration and invasion assay (× 20). Mean ± SD, n = 3. *P < 0.05, **P < 0.01, or ***P < 0.001.

To determine the role of HAS3 protein in NPC cells, endogenous HAS3 expression was depleted in HK1 or FaDu cells by shRNAs expressing lentivirus. Alternatively, a HAS3 cDNA-expressing lentivirus was used to establish gain-of-function models in CNE1 and CNE2 cells. The mRNA expression levels of HAS3 in the gain- or loss-of-function models were measured by qPCR (Figure 4A). And protein levels in lentivirus-infected cells were determined using western blotting (Figure 4B). The effect of HAS3 expression on NPC cells growth, motility and invasiveness was measured. As evidenced by the CCK-8 assay, silencing HAS3 in HK1 and FaDu cells slowed cell growth in vitro, but re-expression of HAS3 in CNE1 and CNE2 NPC cell lines promoted cell proliferation (Figure 4C). We also performed Boyden Chamber migration and invasion assays and demonstrated that genetically silencing HAS3 in HK1 and FaDu cells inhibited cell metastatic and invasiveness (Figure 4D), while re-expression of HAS3 enhanced malignant potential of CNE1 and CNE2 NPC cells in vitro (Figure 4E).

### Loss of HAS3 decreased tumor metastasis in vivo

**Figure 5:**
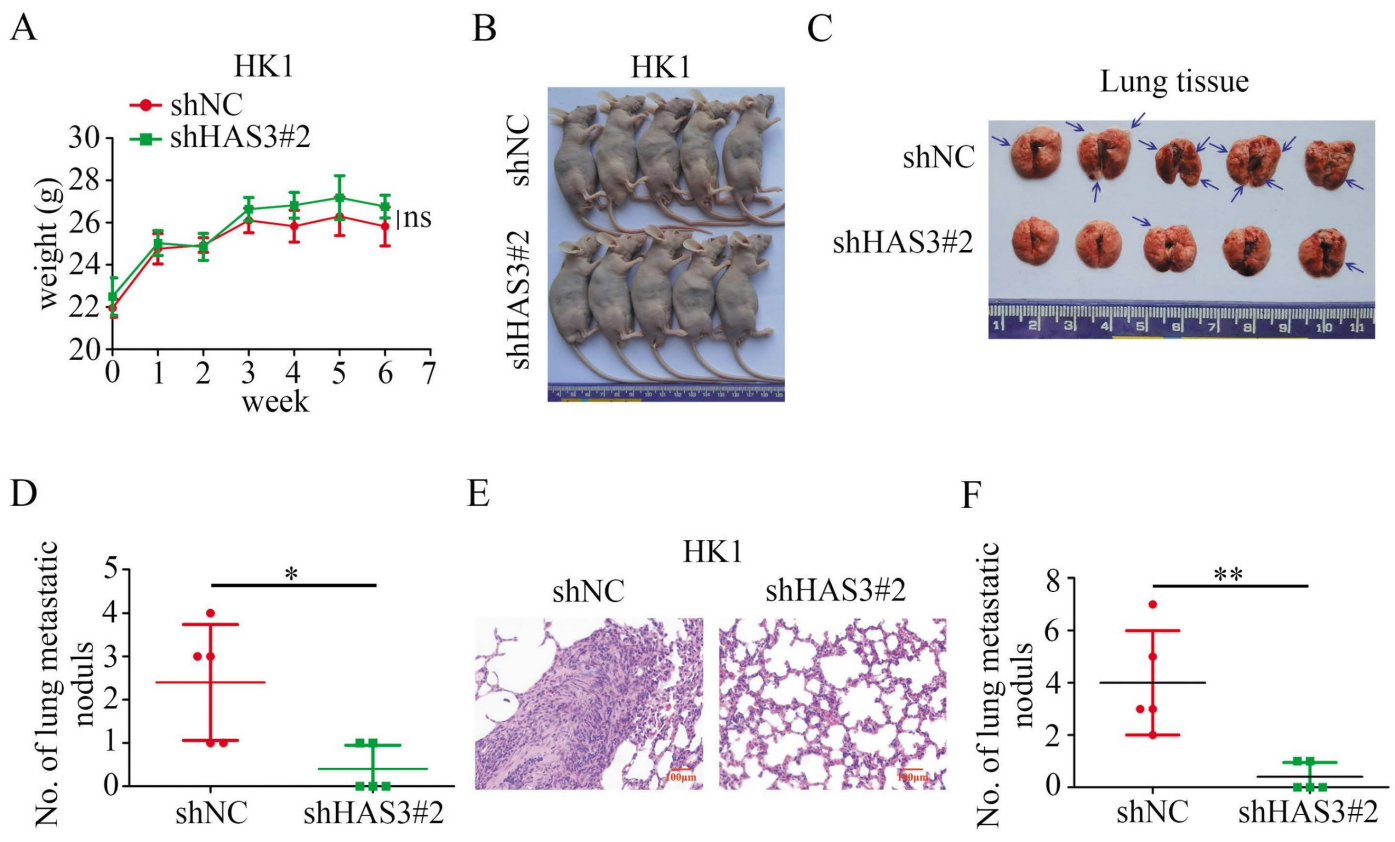
** Loss of HAS3 suppressed NPC cell metastasis in vivo.** (A) Body weight change curve in nude mice after tail vein injection. (B) The macroscopic view of nude mice at the endpoint of experiment. (C) Nude mice metastasis assay showed the macroscopic view of mice lung at 6 weeks after tail vein injection of shNC and shHAS3 NPC HK1 cells. Arrow: metastatic lung nodules. (D) The number of lung metastatic tumor nodules in mice were counted on the surface (n = 5, respectively). (E) Representative H&E staining of mice lung metastatic tumors are shown. (F) H&E staining of metastatic tumors in lung tissues and node counts. In the graph, the scatter diagram shows the number of metastatic nodules in 5 tissue sections from each group. ns, no significance, *P < 0.05, **P < 0.01.

We then asked whether HAS3 promotes NPC cells metastatic potential in vivo. 1 × 10^6^ HK1 cells stably transfected with shHAS3 or empty vector were injected into nude mice through tail vein to evaluate the lung metastatic potential for 6 weeks (Figure 5A and B). Compared with the control group, less and smaller tumor nodules in the lung of shHAS3 groups, indicating that loss of HAS3 suppressed the metastatic ability of HK1 cells (Figure 5C and D). Moreover, tumor formation in lung tissue was examined by H&E staining, which showed that loss of HAS3 inhibited HK1 NPC cell differentiation with a decreased nucleus-to-cytoplasm ratio, but control cells result in severe tumor formation in the lung (Figure 5E and F). These data indicate that HAS3 plays a role as a bona fide tumor promotor in NPC.

### JQ1 and 4-MU inhibited the expression of EGFR and downstream effectors on HK1 cell line

**Figure 6:**
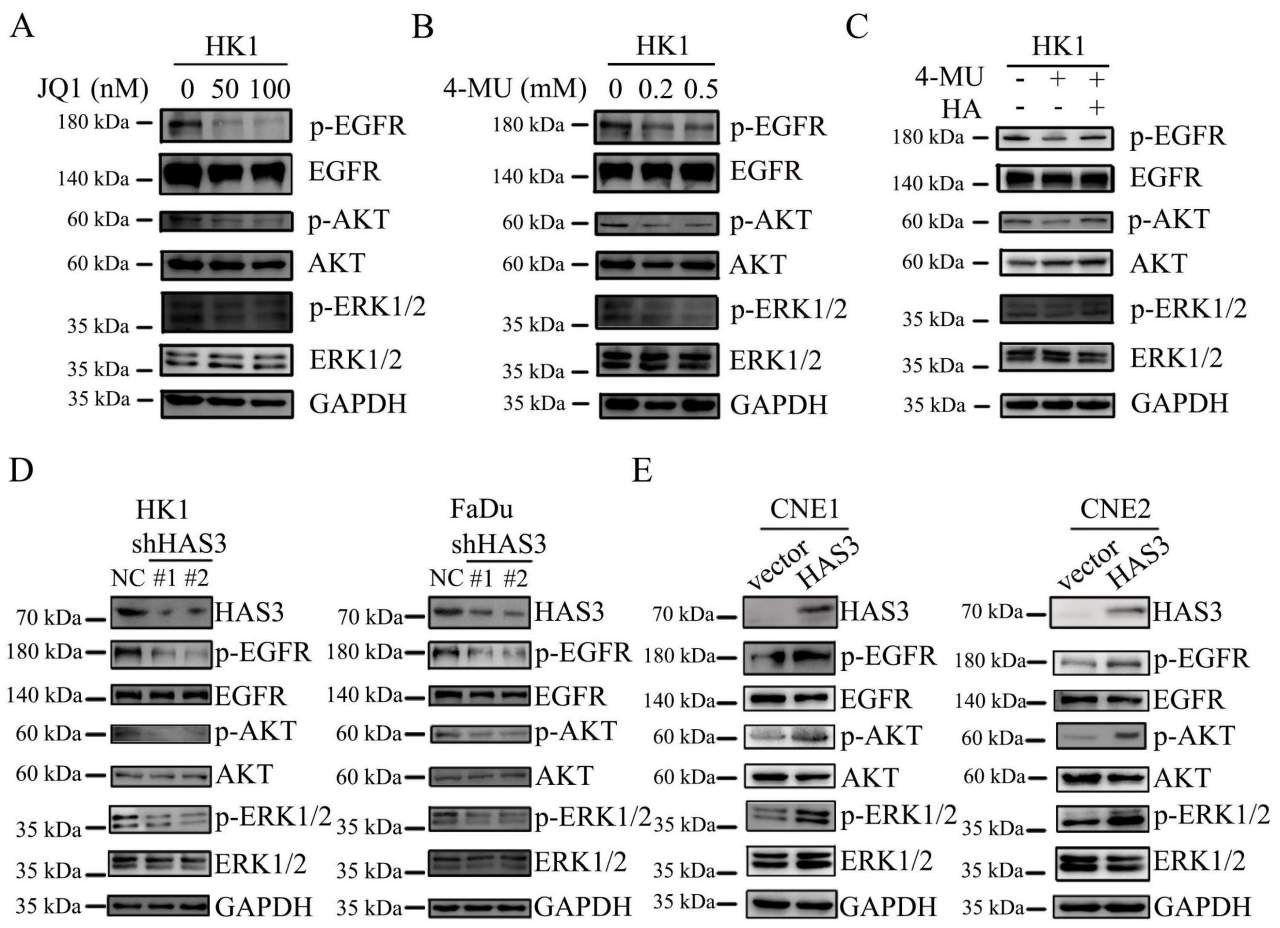
** JQ1, 4-MU and HAS3 have influence on the level of EGFR and downstream effectors expression on NPC cell lines.** (A) p-EGFR, EGFR, p-AKT, AKT, p-ERK1/2, and ERK1/2 expression in HK1 cellular extracts were treated with JQ1 at 50 nM and 100 nM concentration. (B) Western blotting was applied to detect the protein level of p-EGFR, EGFR, p-AKT, AKT, p-ERK1/2, and ERK1/2 after 4-MU treatment at 0.2 mM and 0.5 mM concentration in HK1 cells. (C) Protein expression of p-EGFR, EGFR, p-AKT, AKT, p-ERK1/2, and ERK1/2 were detected by western blotting in HK1 cells treated with or without 4-MU and HA. (D and E) HAS3, p-EGFR, EGFR, p-AKT, AKT, p-ERK1/2, and ERK1/2 protein levels in HAS3-silenced HK1 and FaDu NPC cells (D) and in HAS3 overexpression CNE1 and CNE2 NPC cells (E) were measured by western blotting.

In HK1 cells, inhibition of phosphorylated (p)-EGFR was obtained exposure to JQ1, at a concentration of 50 nM. Concomitant with a downstream effect yields a decrease in p-AKT and p-ERK1/2 protein expression, but JQ1 treatment did not affect the total protein levels of EGFR, AKT and ERK1/2 (Figure 6A).

It has been established that HA promotes EGFR-mediated signaling and EGFR phosphorylation in HNSC 38. Therefore, we utilized HA and HA synthesis inhibitor 4-MU to conduct subsequent experiments. Exposure of the NPC cell line HK1 to 4-MU, at a concentration of 0.2 mM and 0.5 mM resulted in a decrease in p-EGFR expression level. Concomitant with a downstream effect yields a decrease in p-AKT and p-ERK1/2 protein expression (Figure 6B). However, the addition of HA on 4-MU treated HK1 cells had effect on p-EGFR, p-AKT and p-ERK1/2, and restored protein expression (Figure 6C).

### HAS3 promoted EGFR and downstream effectors expression on NPC cell lines

We next examined whether HAS3 expression affected the downstream signaling of EGFR. The p-EGFR, EGFR, p-AKT, AKT, p-ERK1/2 and ERK1/2 protein levels in loss or gain of function of HAS3 NPC cells were measured by western blotting assay. As shown in Figure 6, shRNA-dependent loss of function of HAS3 in HK1 and FaDu cells led to a reduction in the levels of p-EGFR and downstream p-AKT and p-ERK1/2 (Figure 6D). By contrast, HAS3 expression in CNE1 and CNE2 cells resulted in a significant elevation of p-EGFR, p-AKT, and p-ERK1/2 (Figure 6E). Neither loss nor gain of function of HAS3 affected the total protein levels of EGFR, AKT and ERK1/2 (Figure 6D and E).

## Discussion

Our study showed that HAS3 is overexpressed in NPC and driven by SE. High expression of HAS3 promoted malignant progression of NPC in vitro and in vivo. Exogenous HA treatment or HAS3 also activated EGFR/AKT/ERK signaling pathway.

NPC is characterized by high invasion and early metastasis, 70-80% of NPC patients have developed cervical lymph node metastases at the first diagnosis 2-5. Cancer research has been elucidating mechanisms of metastasis for decades 57, 58. However, the underlying mechanism of NPC metastasis is still not clear. Representative characteristics of NPC include comparatively low mutation rate, widespread hypermethylation, high copy number alterations and chromosome abnormalities 6-14. Thus, the exploration of epigenetic regulation is a promising direction in the field of the processes evolve on NPC.

Since in tumor development and progression, SEs often drive oncogene overexpression and may provide an opportunity to identify oncogenes behaviors 19-21. Based on this strategy, in previous work (Jing Cai et al. 2019) 46, we built the SE landscapes in HK1 and C666-1 NPC cells, and identified HAS3 as a SE-driven oncogene from the analyzed data by stitching H3K27ac peaks using the ROSE algorithm. To prove the transcription of HAS3 is associated with SE, JQ1, the wide spectrum inhibitor of SE was used. We found that JQ1 treatment resulted in downregulation of HAS3 in HK1 and FaDu NPC cells in a dose-dependent manner and inhibition of cell proliferation and invasiveness in NPC cells. In addition, results showed that JQ1 treatment inhibited the activation of the EGFR/AKT/ERK pathway in HK1 NPC cells.

In several cancer types, HA is important for tumorigenesis and tumor progression 59, 60. HA synthesis is one of the important mechanisms for HA accumulation, which is associated with HAS family members in tumor 61, 62. It has previously been reported that the expression levels of HAS3 were higher than that of HAS1 and HAS2 in most oral cancers, and HAS3 was more catalytically active in HA synthesis than HAS1 and HAS2 36. In this study, we found that NPC cells expressed high levels of HAS3 mRNA and protein, which is in contrast to the normal nasopharynx epithelium (NPE) cell lines. Inhibition of HA synthesis by HAS inhibitor 4-MU suppressed cells growth and invasion of HK1 and FaDu NPC cells in vitro, whereas HA treatment exerted opposite effects. To some extent, these results are consistent with previous studies on that HA promotes tumor cells proliferation and invasiveness. Furthermore, HAS3 expression correlate to malignant transformation 63 and the overexpression of HAS3 in several cancer types, such as prostate cancer, osteosarcoma and colon carcinoma is known to be associated with higher malignancy or metastasis 64-66. According to our observation, genetically silencing HAS3 in HK1 and FaDu NPC cells attenuated cell proliferation and mobility, while re-expression of HAS3 enhanced malignant potential of CNE1 and CNE2 NPC cells. And the effect of HAS3 overexpression was comparable to that of the enough additional HA supplementation. In addition, loss of HAS3 impaired metastatic potential of HK1 cells in nude mice. Thus, we demonstrated for the first time that HAS3 promotes malignant progression of NPC.

Consistent with the HA-mediated activation of EGFR signaling 38, previous research has demonstrated that HAS3 exerts its biological functions in tumor via activation of EGFR related pathways 36. The mutation of EGFR gene tumorigenic mutation is a cause of NPC 8. And the activation of EGFR signaling pathways promote carcinogenesis, by increasing tumor cell proliferation, migration, angiogenesis and apoptosis inhibition 67. Therefore, in this study, we explored EGFR and its downstream signaling pathways in NPC. Western blotting analysis showed that 4-MU treatment did not affect the total protein levels of EGFR, AKT and ERK, but reduced the phosphorylated protein level of EGFR, AKT and ERK in a dose-dependent manner, respectively. In contrast, exogenous HA treatment activated EGFR/AKT/ERK signaling cascade. Furthermore, the effect of HAS3 on EGFR/AKT/ERK pathway was further investigated in this study through directly knockdown or overexpression of HAS3 expression. And the results showed that loss of HAS3 protein inhibited the expression of the phosphorylation of EGFR, AKT and ERK proteins in HK1 and FaDu cells, but HAS3 overexpression promoted EGFR/AKT/ERK signaling pathway in CNE1 and CNE2 cell lines. In summary, all these results demonstrated that HAS3 expression induces the activation of the EGFR/AKT/ERK pathway in NPC.

In conclusion, we depicted for the first time that HAS3 is driven by SE and overexpressed in NPC. High expression of HAS3 promotes the malignant features of NPC in vitro and in vivo. In addition, this study mechanistically explaining the role of HAS3 in cancer development. Exogenous HA or overexpression of HAS3 promotes activation of the EGFR/AKT/ERK pathway, but HA inhibition or HAS3 silencing attenuates activation of the EGFR/AKT/ERK pathway in NPC. In summary, this study highlights the role of SE-driven oncogene HAS3 which promotes malignant progression of NPC and complements the mechanism and provide a novel approach for identifying oncogenic factors in NPC development.

## Abbreviations

NPC: nasopharyngeal carcinoma
EGFR: epidermal growth factor receptor
SEs: super enhancers
TFs: transcription factors
TEs: typical enhancers
HAS3: hyaluronan synthase 3
ECM: extracellular matrix
HA: hyaluronan
HAS: hyaluronan synthase
EGFR: epidermal growth factor receptor
ESCC: esophageal squamous cell carcinoma
EGF: epidermal growth factor
ROSE: ranking of super-enhancer
qPCR: real-time Reverse-transcription PCR
CCK-8: Cell Counting Kit-8
TCGA: The Cancer Genome Atlas
4-MU: 4-Methylumbelliferone
NPE: normal nasopharynx epithelium
